# Oral Glucose Tolerance Test for Everyone: Affordable, Accessible, and Well-Tolerated Alternatives to Traditional Glucose Liquid

**DOI:** 10.7759/cureus.100007

**Published:** 2025-12-24

**Authors:** Andrew El Alam, Mohamad Fleifel, Abd al latif Awdi, Khaled Abi Farraj, Amal Al Zoghbi, Bertha Maria Nassani, Raoul Al Kassis, Hicham Baba, Soha Bayda, Assem Al Hariri, Arnaud Monier

**Affiliations:** 1 Department of Endocrinology, Lebanese American University Medical Center, Beirut, LBN; 2 Department of Endocrinology, American University of Beirut Medical Center, Beirut, LBN; 3 Department of Endocrinology, Beirut Arab University Medical Center, Beirut, LBN; 4 Department of Internal Medicine and Clinical Immunology, Hospital Hôtel Dieu de France, Beirut, LBN; 5 Department of General Medicine, Lebanese University Faculty of Medicine, Beirut, LBN; 6 Department of Endocrinology, Louis Pasteur Hospital Center, Chartres, FRA

**Keywords:** alternatives, cost effectiveness, diabetes, diagnosis, glucola, ogtt, screening, tolerance

## Abstract

The growing global burden of diabetes requires innovative strategies for accurate and patient-friendly diagnostic methods. The oral glucose tolerance test (OGTT), a long-standing cornerstone in diabetes screening, employs a glucose solution called Glucola. However, the drawbacks of Glucola, including discomfort, side effects, and cost, have fuelled the exploration of alternative substances for OGTT. This comprehensive review article examines the clinical significance, patient acceptance, and cost-effectiveness of these emerging alternatives. An extensive literature search in PubMed and Google Scholar was done for articles published after 1995, but priority was given to articles published after the year 2000. Twelve articles discussing alternatives to Glucola were included in this review. Research consistently indicates that these alternatives offer diagnostic accuracy comparable to that of Glucola while mitigating patient discomfort and reducing financial burdens. The economic aspect of diabetes detection is a key consideration, given the substantial costs associated with Glucola-based testing protocols. By synergizing alternative approaches with patient education, diabetes screening can be made more accessible and cost-effective. The present article reviewed the available clinical data on alternatives explored for oral glucose tolerance testing (OGTT) beyond the use of Glucola, shedding light on their accuracy and tolerance in different population groups.

## Introduction and background

From early insights to the evolution of OGTT

The field of medical practice is witnessing an alarming increase in the prevalence of diabetes mellitus worldwide. In 2024, around 589 million adults are diagnosed with diabetes, and it is estimated to rise to 853 (1 in 8) million by 2050 [1]. In the United States, the number of people with diabetes reached 38.4 million in 2021, representing 11.6% of the US population [2]. Therefore, it is imperative to identify individuals at high risk of developing diabetes [3]. Among these high-risk individuals, prediabetic patients are particularly susceptible [4]. Advancements in medical science have led to rapid progress in diagnostic tools, making the investigation of glucose intolerance an intriguing area of study [5]. One such diagnostic test is the oral glucose tolerance test (OGTT), which dates back to 1917 when a 100 g standard glucose load test was first introduced [6]. Over time, different glucose doses were explored, leading to the adoption of the 75 g dose as the official standard for OGTT by the World Health Organization (WHO) in 1980 [7]. Now, the oral glucose tolerance test (OGTT) has been the mainstay for the detection of prediabetes and type two diabetes for more than a century [6]. According to the World Health Organization (WHO) and the International Diabetes Federation, OGTT was recognized as a sensitive and widely accepted diagnostic test for diabetes, alongside fasting plasma glucose and glycated hemoglobin (HbA₁c) [8]. Their recommendation is to use a 75-gram oral glucose tolerance test (OGTT) measuring fasting and two-hour plasma glucose to detect IGT and IFG [9]. Early detection and detection are of crucial importance since undiagnosed diabetes continues to increase the risk of having diabetes-related complications [10].

OGTT may seem straightforward, involving the oral intake of 75 g of glucose for nonpregnant adults and 1.75 g/kg (up to a maximum of 75 g) for children and adolescents after an overnight fasting period of 8-12 hours [6,11]. Blood sugar levels are measured at the beginning of the study and then again two hours after glucose intake [12]. Normal fasting blood sugar is defined as less than 100 mg/dl, while abnormal fasting or prediabetes falls within the range of 100-125 mg/dl, and diabetes mellitus is indicated by levels above 125 mg/dl [13]. Numerous studies have shown that OGTT is an excellent predictor of diabetes, especially when fasting blood sugar levels are normal [3]. However, despite its apparent simplicity, OGTT is not without complexities and side effects. Reports of nausea, vomiting, abdominal pain, sweating, and headaches have been associated with the Glucola beverage administered during this screening test [5]. Considering the cost and adverse effects of Glucola, researchers have been motivated to explore alternative tests with fewer constraints.

This review article aims to explore the various alternatives used in place of the Glucola liquid. It provides a highlight of other substitutes utilized for OGTT that can be used instead of Glucola, depending on their local presence.

## Review

Materials and methods

The research team used computerized searches for articles published in PubMed and Google Scholar. We collected information from several research studies. Articles published after 1995 and discussing alternatives to traditional glucose liquid were included, but priority was given to articles beyond the year 2000. All study types were included as long as they contributed to the review article without a level of redundancy. The terms used were “alternatives for the Glucola test,” “costs of the Glucola test,” “OGTT and Glucola,” “adverse effects of Glucola,” “uses of Glucola,” and “replacement of the Glucola liquid.” Articles not published in English and not available in full text were excluded. After going through available articles and applying inclusion and exclusion criteria, 12 articles were selected. It should be noted that our article serves as a review of the literature. The objective of this article is to study more tolerated alternatives to Glucola for the oral glucose tolerance test.

The clinical value of oral glucose tolerance testing (OGTT)

OGTT not only helps define high-risk patients but also plays a crucial role in the detection of diabetes [11]. The application of OGTT extends from the diagnosis of borderline diabetes, fasting, and postprandial glucose levels to its use in hypertriglyceridemia, neuropathy, impotence, diabetes-like renal diseases, retinopathy, glycosuria without hyperglycemia, reactive hypoglycemia, acromegaly, and disorders of carbohydrate metabolism [5]. Furthermore, OGTT plays a crucial role in the detection of diabetes during pregnancy [6,14]. It is also an essential tool in the diagnosis of DM in patients with hemoglobinopathies and screening for diabetes related to cystic fibrosis [15,16]. The importance of early detection lies in the fact that diabetes mellitus remains asymptomatic and undiscovered for 9 to 12 years, already having complications when diagnosed [17].

Alternatives tested to the Glucola liquid

Numerous studies have been conducted to explore alternatives to the standard solution used in oral glucose tolerance tests (OGTT) (Table 1).

**Table 1:** Characteristics of the patients studied with different alternatives to Glucola, describing the population studied, the alternative product, the results obtained, and the advantages of the alternative. MMTT: Mixed Meal Tolerance Test, OGTT: Oral Glucose Tolerance Test, GDM: Gestational Diabetes Mellitus, DBGP: Designed Breakfast Glucose Profile, GCT: Glucose Challenge Test, CFRD: Cystic Fibrosis-Related Diabetes, PCOS: Polycystic Ovary Syndrome, BT: Breakfast Test

Population	Alternative Studied	Results	Advantages of The Alternative	Adverse events using Alternative	Reference
13 subjects that have undergone islet transplantation for Type 1 diabetes with stable graft function	MMTT (Mixed meal tolerance test) Containing 55% carbohydrates	Close association between a 90-minute glucose 8.0 mmol / L MMTT and the 120-minute OGTT threshold for the diagnosis of diabetes (OGTT120 ≥11.1 mmol/L)	Has less than half the carbs of a 75g-OGTT, reducing hyperglycemia and minimizing metabolic stress on the islet graft	none	Forbes et al., 2018 [18]
51 Patients considered at high risk of developing GDM	DBGP (Designed breakfast glucose profile) containing 75 g carbohydrates	Satisfactory correlation between current “gold standard” OGTT and the DBGP	Wide availability locally and in community-care clinics. Could easily be premeasured to contain 75 g of carbohydrate	none	Marais et al., 2018 [19]
104 healthy individuals excluding Pregnant women	Ice Cream 73.9 g carbohydrates	Good correlation of 2-hour plasma glucose between both Ice Cream and standard 75-g glucose	Preferred by participants due to its better taste and easier ingestion	none	Chanprasertpinyo et al., 2017 [20]
30 Healthy individuals	New lemon-lime flavored formula (1,000 milligrams of citric acid and 0.03 gram of lime flavor to 75 grams of glucose to a final volume of 300 ml)	No difference in plasma glucose values and OGTT-derived parameters responses to OGTT in comparison to the traditional formula	Higher satisfaction score No alteration of glycemic and insulin surrogate markers response to OGTT	none	Phawinpon et al. 2016 [21]
20 women already screened positive for GDM based up a 50-g 1-hour GCT	10 candy twists equivalent of the 50-g glucola	Candy twists are equally effective screening method for GDM when compared with the standard 50-g glucola drink but result in fewer false-positive screens	Better tolerance Cost effective	none	Racusin et al., 2015 [5]
Cystic Fibrosis	Apple Juice (Sun Cup) 6 containers containing 78 g carbohydrate Grape juice (Sun Cup) 4 containers containing 76 g carbohydrate Orange juice (Sun Cup) 6 containers containing 78 g carbohydrate Soda (Coca-Cola) 2 United States cans (355 mL) containing 78g carbohydrate	Standardized beverage choices that give a glucose load to similar to glucola Values calculated using Nutritionist Pro Version 4.6.0. Axxya Systems. Stafford, Texas, 2011	Better accepted by the patients and their families Increased the screening rate of CFRD in the pediatric clinics	none	Franco et al., 2011 [22]
15 women with a diagnosis of PCOS	Costco chocolate chip muffin and + 8 oz. container of Tropicana orange juice Containing 105 g carbohydrate	equally as effective as the OGTT in diagnosing impaired glucose tolerance, and in fact, may even be more sensitive	Fewer side effects Better tolerance	none	Freeman et al., 2010 [23]
60 Healthy individuals	Mixed Meal Containing 90 g carbohydrate	Strong correlation between the 2 h OGTT and a standardized test meal	Not indicated in the study	none	Meier et al., 2009 [24]
141 pregnant women	Standard breakfast containing 50 g of simple glucose	Concordance of GTT with GCT and BT was 0.429 and 0.432, respectively, and both were statistically significant standard breakfast can be used as alternative to the GCT for assessing carbohydrate intolerance in pregnancy	Better tolerance	none	Eslamian et al., 2006 [25]
19 Healthy, non-obese individuals 20-65 yo	Cookie (1 pack) containing 75 g carbohydrate	Cookie test provided more data compared to liquid glucose	Fewer epigastric side effects and reactive hypoglycemia	none	Harano et al., 2006 [26]
160 pregnant women at 24 to 28 weeks' gestation	Jelly beans 28 jelly beans equivalent of the 50-g glucola	No significant differences were found between 1-hour serum glucose values with 50-g glucose beverage, with jelly beans, frequency of discrepant results, sensitivity, specificity, or predictive value	Better preference fewer side effects	none	Lamar et al., 1999 [27]
157 women between 26 to 30 weeks of gestation	Jelly beans 18 jelly beans	Using a 140 mg/dl threshold, the sensitivity, specificity, and positive predictive value of the cola beverage was 46%, 81%, and 18%. These values at a 120 mg/dl threshold for jelly beans were 54%, 81%, and 20%, respectively	Better tolerance	none	Boyd et al., 1995 [28]

Healthy Individuals

In 2006, Harano et al. explored the use of a cookie containing 75 g of carbohydrates (divided into 85% flour starch and 15% maltose) as a substitute for the conventional solution. Their research included 19 healthy non-obese volunteers and highlighted the superior tolerance of the cookie used, with fewer side effects such as epigastric discomfort and hypoglycemia, while providing more comprehensive information [26]. The investigations by Phawinpon et al. in 2016 and Chanprasertpinyo et al. in 2017, which included 30 and 141 healthy participants, respectively, introduced two alternatives to the typical 75 g glucose solution used in OGTT. The former group tested a non-carbonated lemon-lime drink with 75 g of glucose (a mixture of 1000 mg of citric acid, 0.03 g of lime flavor, and 75 g of anhydrous glucose, resulting in a solution of 300 ml), while the latter examined ice cream containing 73.9 g of glucose. Both studies signaled an increase in patient tolerance and acceptance. Therefore, these methods can serve as comparable replacements for traditional solutions, with a minor sensitivity observed in the ice cream alternative that requires validation in a more extensive population before being integrated into clinical practice [20,21].

Furthermore, the 2008 study by Meier et al., which examined 60 subjects (differentiated by their glucose tolerance status: normal, impaired, or diabetic), established a strong correlation between a mixed meal of a total of 820 kcal (with 360 kcal only from carbohydrates) and the 75 g oral glucose challenge solution with an equal 360 kcal content [24].

Pregnant Women

Regarding pregnant women, during 2006, Eslamian et al. carried out a comprehensive study involving 141 pregnant women to compare the effectiveness of a standard breakfast containing 50 g of simple glucose with the prevalent glucose challenge test and the glucose tolerance test using 50 g and 100 g of glucose, respectively, both established tools for screening gestational diabetes (GDM). The study results showed significant congruence among the three tests, advocating for the adoption of the standardized breakfast as a screening alternative that demonstrated improved tolerance and compliance compared to the conventional solutions [25]. Comparable results were observed in a 2018 research study by Marais et al. that used a designed breakfast glucose profile (DBGP) comprising 75 g of glucose to detect gestational diabetes (GDM) in 51 high-risk individuals for GDM. Their findings suggested that DBGP could be a feasible alternative to the typical solutions used in OGTT [19]. Within the scope of GDM, Racusin et al.’s 2015 research involved 20 pregnant participants (who had already tested positive with the Glucola 50 g glucose challenge test), using candy twists with a matching glucose content for comparison. The study discovered that the candy twist method was just as reliable, resulted in fewer false positive outcomes, offered better patient tolerance, and presented a more cost-effective option [5].

A clinical trial attempting to test the tolerance and diagnostic efficacy of jelly beans as a replacement for a 50 g solution. The sensitivity, specificity, and positive predictive value of using jelly beans were all better than those of the 50 g cola beverage. Patients also reported better tolerance to jelly beans and fewer side effects compared to the period after the cola beverage. Concluding that jelly beans can serve as a substitute for the 50 g glucose beverage as a sugar source during gestational diabetes mellitus screening [28].

Another similar study of 136 participants compared patients randomly assigned to 50 g of glucose equivalent of jelly beans (28 jelly beans) vs. 50 g of cola beverage and showed no significant differences when using both methods. However, the use of jelly beans was associated with fewer side effects and was preferred by 76% of the participants. Therefore, jelly beans could be an effective replacement for a 50 g cola beverage [27].

Others

Polycystic ovary syndrome (PCOS), being an independent risk factor for insulin resistance, requires the use of OGTT, which serves as a key tool in evaluating patients with PCOS. Freeman et al. explored an alternative test meal for 15 patients diagnosed with PCOS, consisting of a large Costco chocolate chip muffin and an 8-oz portion of Tropicana orange juice, combining 105 g of carbohydrates, 70 g of which was sucrose. The findings suggest that this muffin-based test paralleled OGTT in its diagnostic accuracy for impaired glucose tolerance and potentially provided increased sensitivity, with markedly reduced side effects and better overall tolerance [23].

Lastly, in their research, Forbes et al. analyzed 13 subjects with stable graft function post-islet transplantation for type 1 diabetes. Their focus was on comparing the efficacy of a mixed meal test (measure glucose at 90 minutes with a threshold of > 8.0 mmol/L) against the conventional 75 g OGTT (measure glucose at 120 minutes with a threshold of > 11.1 mmol/L). Their findings accentuated a clear association between the two diagnostic approaches. Furthermore, MMT was identified as a powerful tool to detect candidates who would benefit from intensified intervention and supportive measures to maintain graft viability after post-islet transplantation. A significant advantage of MMT was its reduced carbohydrate content compared to 75 g of OGTT, which translates to a lower risk of hyperglycemia and decreased metabolic stress on the islet graft [18].

Limitations of Glucola in OGTT

Problem of Intolerance

The glucose tolerance test is usually given in the form of a viscous and sweet liquid. This beverage, known as Glucola, containing 37% glucose, 11% maltose, and 10% triose and can cause several well-known side effects, such as vomiting, sweating, abdominal pain, and bloating. Due to its rapid absorption and high osmolarity, it can cause reactive hypoglycemia. It is worth noting that a considerable percentage of patients, ranging from 10% to 15%, may refuse to undergo this test or experience vomiting. As a result, some people may not receive proper screening for the potentially significant condition [23,28,29]. It is important to remember that the body's response to the OGTT is not physiological, producing an insulin and glucagon response that is different from more physiological stimulation, such as a meal [30].

Pregnancy After Bariatric Surgery

Due to the success of bariatric surgery in weight loss, we are experiencing an increase in the number of obese women of childbearing age who undergo bariatric surgery. OGTT remains a crucial test in these women, despite intolerance to Glucola liquid and fluctuations in blood sugar levels. Several studies have shown strong evidence of reactive hypoglycemia during the oral glucose tolerance test (OGTT) in approximately 50-58% of pregnant women who have undergone bariatric surgery [31]. Furthermore, a recent investigation revealed that the probability of experiencing reactive hypoglycemia was markedly higher in women who had undergone Roux-en-Y gastric bypass (RYGB), reaching 83%, compared to those who had previously undergone sleeve gastrectomy (54%) or adjustable gastric banding (12%). Reactive hypoglycemia in these women showed higher rates of small-for-gestational-age infants, reaching 11.9% [32].

Bariatric Surgery in the General Population

In patients after bariatric surgery, several side effects of Glucola were observed, ranging from nausea, dizziness, and weakness to hypoglycemia, tachycardia, and tremor in some cases. This intolerance to OGTT in patients after bariatric surgery is caused by early and late dumping after ingesting the glucose load [33,34].

Cystic Fibrosis and the Need to Screen for Diabetes at a Young Age

Because CFRD often goes unnoticed, the recommendations of the Cystic Fibrosis Foundation (CFF), the American Diabetes Association (ADA), and the Pediatric Endocrine Society (PES) suggest conducting a yearly screening for CFRD beginning at the age of 10. This screening should involve an oral glucose tolerance test (OGTT) of 2 hours following the World Health Organization protocol. Glucola is not tolerated by many young children, leading to their refusal to drink the needed amount, rendering the assessment challenging. As a solution to this problem, other common beverages with similar glucose content were standardized as Glucola (Table 1). The use of Glucola alternatives was much more tolerated in these children, who already suffer from delayed gastric emptying. Not only were they preferred by children and their parents, but they also led to an increase in the screening rate in pediatric CF clinics [22].

Cost-Effectiveness

Regarding costs, alternatives are typically more cost-effective than using the oral glucose solution. Several studies suggest that the oral glucose solution can be quite expensive, with reported prices ranging from approximately $5.20 to $6 per unit. Furthermore, certain references indicate that the expenses could potentially be even higher than these figures [26,35].

According to Racusin et al., at their institution, the expense associated with a 3-hour GTT, which includes blood draws, amounts to $100. However, if they had chosen an alternative approach using candy twists for their study population of 20 subjects, they could have saved at least $948.80. These price points are of significance in nations where wages are modest, and some of these countries have a minimum wage as low as $16 per month [36].

However, most of the alternatives were much cheaper, in some studies reaching as low as $0.50 each [23].

Importance of Education

Whether using Glucola liquid or alternatives, patient education remains crucial in increasing the acceptance rate for screening. A cross-sectional study that included 152 women between 24 and 32 weeks evaluated their knowledge and anxiety during OGTT and showed that 40% of the patients did not know why they were doing the test [37]. Of these women, 48% had high anxiety levels, and 47% were very disturbed by this test. Another study involving 385 pregnant women investigated how education influenced the results of the oral glucose tolerance test (OGTT). The findings revealed that 79.3% of women who received education about the test opted to undergo it. On the contrary, only 38.5% of the participants in the control group chose to take the test. Thus, education plays an important role in the acceptance of OGTT [38].

Limitations of This Review

This review has several limitations that we should consider. First, glycemia can vary widely after ingestion of equal amounts of carbohydrates from different food sources, due to differences in glycemic index, glycemic load, and macronutrient composition. Therefore, alternatives to the OGTT with the same carbohydrate amount may still produce different glycemia levels [39,40]. Second, this review doesn’t follow a systematic search strategy, which may introduce a risk for selection bias. Finally, only English-language studies were included, which may have excluded articles published in another language.

## Conclusions

This article highlights the need for more tolerable and cost-effective alternatives to Glucola for OGTT. Various substances have been studied that can serve as a replacement for the standard solution. These studies showed promising results in terms of tolerance, diagnostic accuracy, and cost-effectiveness. However, randomized clinical trials with larger sample sizes are needed to validate the results' accuracy and reproducibility and to address the regulatory barriers that prevent current clinical application. Backed by patient education, these alternatives help physicians address the specific challenges that patients face and ensure an optimal diagnostic environment.
